# Supplementary material for: Spectrum of Genes for Non-GJB2-Related Non-Syndromic Hearing Loss in the Russian Population Revealed by a Targeted Deafness Gene Panel
Source: Int J Mol Sci. 2022 Dec 12;23(24):15748. doi: 10.3390/ijms232415748 (PMC9779600; doi:10.3390/ijms232415748)
Supplement: Supplementary file 1 [file ijms-23-15748-s001.zip › ijms-2036179 suppl. tableS1.pdf]

Table S1. The genotypes identified in this study among Russian patients carrying pathogenic or probably pathogenic gene variants.

Gene	Genotype		Effect		ClinVar ID, The ClinVar accession numbers [b]		Type of Inheritance	Number of Patients	Phenotype
	Allele 1	Allele 2	Allele 1	Allele 2	Allele 1	Allele 2			
ADGRV1	**c.6610C>T**	**c.10198C>T**	**p.Gln2204***	**p.Gln3400***	**this study, SCV002754430**	**this study, SCV002754431**	AR	1	SN NSHL
MYO15A	c.6046+1G>A	**c.8910del**	splice	**p.Val2971fs*63**	VCV000164536	**this study, SCV002754432**	AR	1	SN NSHL
MYO7A	**c.1738_1745del**	c.5573T>C	**p.Val581Leufs*28**	p.Leu1858Pro	**this study, SCV002754433**	VCV000043288	AR	1	SN NSHL
MYO7A	c.2558G>A	wt	p.Arg853His	wt	VCV000043186,	-	AD	1	SN NSHL
MYO7A	c.2557C>T	wt	p.Arg853Cys	wt	VCV001333565	-	AD	1	SN NSHL
MYO7A	c.3262C>T	**c.3893G>A**	p.Gln1088*	**p.Gly1298Glu**	VCV000371695	**this study, SCV002754434**	AR	1	SN NSHL
MYO7A	**c.3612_3615del**	**c.4528G>A**	**p.Ser1205Argfs*26**	**p.Glu1510Lys**	**this study,** SCV002754435	**this study, SCV002754436**	AR	1	SN NSHL
OTOA	**c.562_569dup**	g.(?_21624041)_(21730798_?)del	p.Phe191fs*48	del(OTOA)	**this study, SCV002754437**	VCV000003561	AR	1	SN NSHL
OTOF	**c.2656del**	**c.2656del**	**p.Val886Serfs*114**	**p.Val886Serfs*114**	**this study[c], SCV002754438**	**this study[c], SCV002754438**	AR	1	SN NSHL+AN
OTOF	c.4799+1G>A	c.3192C>G	splice	p.Tyr1064*	VCV000164841	SCV002754442	AR	1	SN NSHL+AN
OTOF	**c.5169_5170del**	**c.4903A>T**	**p.Ile1724Leufs*19**	**p.Arg1635***	**this study[c], SCV002754439**	**this study[c], SCV002754440**	AR	1	SN NSHL+AN
OTOF	**c.4903A>T**	**c.2214+5G>C**	**p.Arg1635***	**splice**	**this study[c],**	**this study[c],**	AR	1	SN

					**SCV002754440**	**SCV002754441**			NSHL+AN
POU3F4	c.916C>T	-	p.Gln306*	-	SCV002754444	-	XLR	1	SN NSHL
POU3F4	**c.983A>G**	-	**p.Asn328Ser**	-	**this study, SCV002754443**	-	XLR	1	SN NSHL
PTPRQ	**c.1291C>T**	c.2726del	**p.Arg431***	p.Glu909Glyfs*15	**this study, SCV002754445**	SCV002754446	AR	1	SN NSHL
SLC26A4	c.85G>C	**c.107A>C**	p.Glu29Gln	**p.His36Pro**	VCV000004839	**this study, SCV002754447**	AR	2	SN NSHL,SN NSHL+EVA
SLC26A4	c.1262A>G	**c.107A>C**	p.Gln421Arg	**p.His36Pro**	VCV000430229	**this study, SCV002754447**	AR	1	SN NSHL
SLC26A4	c.1001G>T	**c.208C>T**	p.Gly334Val (splice)	**p.Pro70Ser**	VCV000189039	**this study, SCV002754448**	AR	1	SN NSHL+EVA
SLC26A4	c.1001G>T	c.1001G>T	p.Gly334Val, HC	p.Gly334Val (splice)	VCV000189039	VCV000189039	AR	1	SN NSHL
SLC26A4	c.1229C>T	c.317C>A	p.Thr410Met	p.Ala106Asp	VCV000043498	VCV001065204	AR	1	SN NSHL+EVA
STRC	c.2171_2174del	g.(?_43890333)_ (43940887_?)del	p.Val724Glyfs*6	del(STRC-CKMT1B - CATSPER2)	VCV000004344	VCV000688049	AR	3	SN NSHL
STRC	c.2171_2174del	**g.(?_43906612)_(43906674_)?del**	p.Val724Glyfs*6	**delSTRC,ex5**	VCV000004344	**this study[c], SCV002754455**	AR	1	SN NSHL
STRC	g.(?_43890333)_ (43940887_?)del	g.(?_43890333)_ (43940887_?)del	del(STRC-CKMT1B - CATSPER2)	del(STRC-CKMT1B - CATSPER2)	VCV000688049	VCV000688049	AR	5	SN NSHL
STRC	g.(?_43890333)_ (43940887_?)del	g.(?_43890333)_ (43897714_?)del	del(STRC-CKMT1B - CATSPER2)	del(STRC-CKMT1B)	VCV000688049	SCV002754456	AR	2	SN NSHL
STRC	g.(?_43890333)_ (43940887_?)del	g.(?_43890333)_ (43893072_?)del	del(STRC-CKMT1B - CATSPER2)	delSTRC,ex25	VCV000688049	SCV002754457	AR	1	SN NSHL

STRC	c.4402C>T	g.(?_43890333)_ (43940887_?)del	p.Arg1468*	del(STRC-CKMT1B - CATSPER2)	VCV000179758	VCV000688049	AR	1	SN NSHL
STRC	c.4057C>T	g.(?_43890333)_ (43940887_?)del	p.Gln1353*	del(STRC-CKMT1B - CATSPER2)	VCV000242391	VCV000688049	AR	1	SN NSHL
TECTA	c.5597C>T	wt	p.Thr1866Met	wt	VCV000236058	-	AD	1	SN NSHL
TECTA	c.1756C>T	**c.2458A>T**	p.Arg586*	**p.Lys820***	**VCV001704157**	**this study, SCV002754449**	AR	1	SN NSHL
TMC1	**c.1750C>T**	c.1250G>A	**p.Gln584***	p.Gly417Glu	**this study, SCV002754450**	SCV002754451	AR	1	SN NSHL
TMPRSS3	c.413C>A	c.208del	p.Ala138Glu	p.His70fs*19	VCV000046119	VCV000165492	AR	1	SN NSHL
TMPRSS3	c.310G>A	c.46C>T	p.Glu104Lys	p.Arg16*	VCV000504522	VCV001210028	AR	1	SN NSHL
USH2A	c.11864G>A	c.11864G>A	p.Trp3955*	p.Trp3955*	VCV000002357	VCV000002357	AR	2	SN NSHL
USH2A	c.11864G>A	c.14365C>T	p.Trp3955*	p.Gln4789*	VCV000002357	VCV000816987	AR	1	SN NSHL
USH2A	c.11864G>A	c.1606T>C	p.Trp3955*	p.Cys536Arg	VCV000002357	VCV000048471	AR	1	SN NSHL
USH2A	c.12234_12235del	c.12234_12235del	p.Asn4079Trpfs*19	p.Asn4079Trpfs*19	VCV000096665	VCV000096665	AR	1	SN NSHL
USH2A	c.11864G>A	g.(?_216270461)_ (216260162_?)del	p.Trp3955*	del*USH2A*,ex22-24.	VCV000002357	SCV002754454	AR	1	SN NSHL
USH2A	c.11864G>A	**g.(?_216108034)_(216108100_?)del**	p.Trp3955*	**del*USH2A*,ex38**	VCV000002357	**this study[c], SCV002754452**	AR	1	SN NSHL
USH2A	c.2610C>A	**g. (?_216462679)_(216462739_?)del**	p.Cys870*	**del*USH2A*,ex11**	VCV000557167	**this study[c], SCV002754453**	AR	1	SN NSHL

Bold font indicates the variants identified for the first time; a—designations are given in accordance with the ACMG recommendations for the interpretation and classification of nucleotide sequence variants [21,22]; b—"VCV-" corresponds to the variant description identification numbers (ClinVarID) in the ClinVar database, —"SCV-" corresponds to the variant accession numbers in the ClinVar database; "rs-" in the dbSNP database; c—variants identified in the same patients are presented in the articles by Markova et al. and Lalayants et al. [14-16]; inheritance type:

AD—autosomal dominant; AR—autosomal recessive; XLR—X-linked recessive; SN NSHL—sensorineural NSHL; SN NSHL+EVA—SN NSHL with enlarged vestibular aqueduct; SN NSHL+AN—SN NSHL with auditory neuropathy.
